# The role of staging laparoscopy in pancreatic adenocarcinoma and its effect on patients’ survival

**DOI:** 10.1186/s12957-022-02803-y

**Published:** 2022-10-11

**Authors:** Maxwell A. Jambor, Amir Ashrafizadeh, Christopher B. Nahm, Stephen J. Clarke, Nick Pavlakis, Andrew Kneebone, George Hruby, Anthony J. Gill, Anubhav Mittal, Jaswinder S. Samra

**Affiliations:** 1 Upper Gastrointestinal Surgical Unit, Royal North Shore Hospital, Sydney, NSW Australia; 2 Sydney Medical School, The University of Sydney, Camperdown, NSW Australia; 3 Department of Pancreatic Hepatobiliary Surgery, Royal North Shore Hospital, St Leonards, NSW 2065 Australia; 4 Sydney Vital, Sydney, NSW Australia; 5 Bill Walsh Translational Cancer Research Laboratory, Kolling Institute, St Leonards, NSW Australia; 6 Department of Hepatopancreatobiliary Surgery, Westmead Hospital, Sydney, NSW Australia; 7 Australian Pancreatic Centre, Sydney, NSW Australia; 8 Cancer Diagnosis and Pathology Group, Kolling Institute of Medical Research, Royal North Shore Hospital, St Leonards, NSW Australia; 9 Department of Medical Oncology, Royal North Shore Hospital, Sydney, NSW Australia; 10 Northern Sydney Cancer Centre, Radiation Oncology Department, Northern Sydney Cancer Centre, Royal North Shore Hospital, St Leonards, NSW Australia; 11 NSW Health Pathology, Department of Anatomical Pathology, Royal North Shore Hospital, St Leonards, NSW Australia

**Keywords:** Staging laparoscopy, Pancreatic disorders, Pancreatic surgery, Survival

## Abstract

**Background:**

Prompt and accurate staging of pancreatic cancer is essential to distinguish patients to benefit from resection with curative intent and those with unresectable disease. A staging laparoscopy is used preoperatively to identify macroscopic or occult metastases not identified on imaging. This single-institution study aims to evaluate the role of staging laparoscopy in patients with pancreatic adenocarcinoma and its effect on overall survival.

**Method:**

Clinicopathologic data were evaluated for all patients undergoing staging laparoscopy for pancreatic adenocarcinoma from July 2014 to December 2019. The study identified 155 patients eligible for analysis. All patients were followed for at least 2 years. Clinical backgrounds, survival curves and prognostic factors were investigated.

**Results:**

Resectability status among the cohort was 62 (40%) upfront resectable, 53 (34%) borderline resectable and 40 (26%) locally advanced disease. The median age was 69, with 44% male patients. Median CA19-9 value was 125 kU/L, and median CA125 value was 22 kU/L. Staging laparoscopy resulted in upstaging nine (15%) upfront resectable patients, five (9%) borderline resectable patients and ten (25%) locally advanced patients. There was positive cytology in 19 (12%), peritoneal deposits in six (4%) and peritoneal liver deposits in seven (5%) patients. Overall, the number needed to treat (NNT) to avoid an unnecessary laparotomy was eight patients.

**Conclusion:**

Staging laparoscopy continues to be a valuable investigation of pancreatic adenocarcinoma. In this institution, one in every eight patients undergoing a staging laparoscopy was upstaged to metastatic disease, thus avoiding an unnecessary laparotomy or a non-curative resection.

## Background

Pancreatic cancer which is predominantly pancreatic ductal adenocarcinoma (PDAC) is the eighth most commonly diagnosed cancer in Australia [1]. The 5-year survival rates for pancreatic cancer are among the lowest of all cancers in Australia (11.2% for men and 11.8% for women) [1]. Prompt and accurate staging of pancreatic cancer is essential to distinguish patients to benefit from resection with curative intent and those with unresectable disease. A staging laparoscopy is used preoperatively to identify macroscopic or occult metastases that were not identified on imaging and thus is used to exclude patients from futile radical surgery.

Staging PDAC is primarily achieved with high-quality contrast-enhanced computed tomography (CT) imaging (sensitivity 89–91%, specificity 85–90%) [2]. High-quality multiphase imaging allows for enhanced visualisation of the surrounding vasculature which can be used to categorise PDAC into upfront resectable, borderline, locally advanced (LA) disease or metastatic disease. National Comprehensive Cancer Network (NCCN) guidelines recommend positron emission tomography (PET) as an adjunct modality in patients at high risk of metastatic disease, such as those with borderline resectable disease, markedly elevated CA19-9, large primary tumours, large regional nodes or very symptomatic presentations [3].

Staging laparoscopy is a minimally invasive procedure used to assess for macroscopic or occult metastases for tumours of the abdomen. These cancers include oesophageal, gastric, pancreatic and periampullary, hepatic and biliary tract [4]. Staging laparoscopy potentially avoids unnecessary laparotomy due to the finding of radiographically occult metastatic disease and assists in further staging of the pancreatic cancer with identifying neoplastic cells in peritoneal fluid cytology.

First developed in 1982, staging laparoscopy carried a positive result of 31–51% [5]. However, with modern diagnostic imaging modalities such as CT and PET, the routine use of staging laparoscopy in modern practice has become controversial. The NCCN guidelines suggest staging laparoscopy to be considered in higher risk pancreas cancer patients with upfront resectable disease, for example markedly elevated serum CA19-9, and for patients with borderline resectable disease before administration of neoadjuvant therapy[3].

Given the significant improvements in the quality of imaging and its evaluation, we undertook this single institution study to examine the impact of the routine addition of staging laparoscopy in the treatment of patients with potentially operable PDAC.

## Methods

### Study design

This study is a retrospective analysis of a prospectively maintained database in a single high-volume pancreatic surgery unit in Sydney, Australia. Ethical approval and consent were obtained from the Northern Sydney Local Health District Human Resources Ethics Council (re:HREC/16/HAWKE/105).

### Patients

All patients who underwent staging laparoscopy for pancreatic adenocarcinoma at the Royal North Shore Pancreatic Surgery Unit from July 2014 to January 2020 were included in this study. Only patients with histologically proven pancreatic ductal adenocarcinoma were included in the cohort. Omissions included patients who had a confirmed cholangiocarcinoma, ampullary cancer or known metastatic neoplasm including metastases to the pancreas. Staging included a CT chest, abdomen and pelvis, and tumour resectability was determined by triple-phase CT scan with pancreatic protocol. All patients were followed for at least 2 years from the time of the staging laparoscopy.

In this unit, staging laparoscopy is performed in all patients with malignant pancreatic tumours who were deemed to have resectable disease. This is determined by fortnightly PDAC-specific multidisciplinary team (MDT) review with case discussion at diagnosis and further discussion following treatment response. Treatment consists of either folfirinox or gemcitabine with albumin-bound paclitaxel chemotherapy agents, while palliative therapy consists of gemcitabine with albumin-bound paclitaxel or solely gemcitabine chemotherapy agents. Patients who show a good response to chemotherapy which is defined as shrinkage or lack of progression of tumour size, resectability of the tumour as determined at MDT review and no evidence of metastatic disease on post chemotherapy imaging were restaged including a repeat staging laparoscopy. Additional factors including a significant drop in either serum CA19-9 or ^18^FDG-PET maximum standardised uptake values (SUVmax) were also considered. Surgical resection is undertaken in those considered at MDT to have a resectable primary tumour with no evidence of metastatic disease on restaging.

### Perioperative variables

Patient demographics including age, sex and the presence of diabetes or jaundice were extracted from the prospectively collected pancreatic surgery database. Tumour markers including preoperative serum CA19-9 and CA125 were extracted from the electronic medical records. CT scan and PET results conducted at the time of diagnosis were reviewed for evidence of metastatic disease and to clarify disease resectability.

### Outcomes definition

A positive macroscopic staging laparoscopy is defined if an identifiable tissue sample collected during the procedure is found to contain malignant cells on histopathological analysis. A positive microscopic staging laparoscopy is defined as peritoneal washings which have shown malignant cells on cytology. The pathological distinction between ‘atypical cells suspicious but not diagnostic of cancer’ and a definitive diagnosis of carcinoma may be subtle and is often dependent on the experience of the individual reporting pathologists. We used standard morphological criteria as suggested by the World Health Organization 2019 classification [6] to make this distinction. If there was any doubt, cases were referred to a specialist pancreaticobiliary pathologist. Immunohistochemistry for epithelial markers (useful for the distinction between atypical reactive mesothelial cells and well-differentiated adenocarcinoma cells) was not performed routinely on all cases; rather, it was performed only on selected cases at the discretion of the reporting pathologists — that is those cases where the degree of cytological atypia was mild and there were sufficient atypical cells in the cell block for testing.

### Laparoscopy technique

After establishing pneumoperitoneum through a 10 mm trocar inserted at the umbilical area, a 30-degree laparoscope is inserted, and two additional 5-mm ports are placed. Firstly, the liver and parietal peritoneum are inspected closely examining for the presence of nodular lesions. Secondly, a small bowel run is performed, closely examining the mesentery to identify any further nodules. Lastly, peritoneal lavage is performed with approximately 500 mL of normal saline. The fluid is aspirated and sent for peritoneal cytology. Washings are performed regardless of the presence or absence of macroscopic disease at laparoscopy.

### Statistical analysis

Statistical analysis was performed using GraphPad Prism 9. Spearman’s Rho test and Mantel-Cox test were used for analysis. Significance was defined as *P* < 0.05. Ethical approval was obtained from the Northern Sydney Local Health District Human Research Ethics Council.

## Results

From the 3rd of July 2014 to the 20th of December 2019, 155 staging laparoscopies were performed at this unit for upfront, borderline or LA pancreatic ductal adenocarcinoma. The median age was 69 (47–88), with 44% male patients. Median CA19-9 value was 125 kU/L, and median CA125 value was 22 kU/L (Table 1). The median follow-up time for patients reported alive was 41 months. Spearman correlation analysis showed no correlation among tumour location, tumour size, serum CA19-9, serum CA125 or SUVmax on ^18^FDG-PET scan and the outcome of the staging laparoscopy (Table 2).

**Table 1:** Patient demographics

Factor	Number or median *N* = 155	% or range
Age	69	47–88
Male/female	68/87	44%/56%
**CA19-9**	125	31–845 (IQR)
**CA125**	22	12–42 (IQR)
**Diabetes**	31	20%
**Jaundice**	82	53%
**Tumour size**		
T1	24	15%
T2	89	57%
T3	26	17%
Indeterminate	16	10%
**Tumour location**		
Head	121	78%
Neck	29	19%
Body	3	2%
Tail	2	1%
**PET SUVmax**		
< 5	31	20%
5–10	61	39%
> 10	17	11%
PET not done	46	30%
**Neoadjuvant chemotherapy**	79	51%
**Tumour resectability**		
Upfront	62	40%
Borderline	53	34%
Locally advanced	40	26%
**Procedure**		
PD	81	52%
DP	13	8%
TP	4	3%

*PD* Pancreaticoduodenectomy, *DP* Distal pancreatectomy, *TP* Total pancreatectomy

**Table 2:** Correlation of tumour location, tumour size, CA19-9 and SUVmax values and staging laparoscopy outcome

Spearman’s correlation	Tumour location	Tumour size	CA19-9	CA125	SUVmax
*R*-value	0.065	0.067	0.155	0.151	0.035
*P*-value	0.423	0.436	0.064	0.253	0.715

### Outcome measures of staging laparoscopy

Staging laparoscopy resulted in upstaging of nine upfront resectable patients (14.5%), five borderline resectable patients (9.4%) and ten patients with LA tumours (25.0%) (Table 3). There was positive cytology in 19 patients (12.2%), peritoneal deposits in six patients (3.9%) and peritoneal liver deposits in seven patients (4.5%). Eleven patients had positive peritoneal cytology for metastasis without any macroscopic evidence of disease (7.1%).

**Table 3:** Staging laparoscopy results according to tumour resectability on CT and PET imaging

**Tumour**	**Number**	**Positive lap**	**Macroscopic**	**Cytology only**
Upfront resectable	62	9	7	2
Borderline resectable	53	5	3	2
Locally advanced	40	10	3	7
All patients	155	24	13	11

Of the nine upfront resectable patients with positive staging laparoscopy, one patient with positive macroscopic disease and positive cytology had a good response to chemotherapy and a subsequent negative staging laparoscopy followed by curative intent resection. The remaining eight patients commenced palliative chemotherapy, subsequently developing progression of disease. The one patient that underwent resection lived for 32 months compared with a median survival of 8 months in the other eight upfront disease patients with positive staging laparoscopy that did not have resection.

Among 53 patients with borderline resectable tumours with no metastatic disease on imaging, five had a positive staging laparoscopy (9.4%), with three patients showing macroscopic disease and the remaining two only having microscopic disease on peritoneal wash cytology. All three patients with macroscopic disease underwent chemotherapy. Of these, two had a negative repeat staging laparoscopy and underwent a curative resection. The two patients with persisting microscopic disease on staging laparoscopy had rapid disease progression and passed away in 11 and 17 months, respectively. Of the two patients that underwent resection, one survived 17 months, while the other patient is still alive after 82 months, compared to a median of 11 months for the three patients that did undergo resection.

Among 40 patients with LA disease, 10 had a positive staging laparoscopy (25.0%). Three had macroscopic metastasis, one with negative cytology and two with positive microscopic disease on cytology. Seven patients with LA disease had only positive microscopic disease on cytology with no macroscopic metastasis (Table 3). All seven underwent chemotherapy with follow-up review and repeat imaging. Two patients had a favourable response to treatment, and after MDT discussion, they underwent a repeat staging laparoscopy, both of which did not demonstrate macroscopic or microscopic disease so underwent curative resection. These patients had a survival of 37 and 10 months, respectively, compared to a median of 4.5 months for the eight patients that did not have a resection.

Combining upfront, borderline and LA disease, staging laparoscopy resulted in 24 of the 155 patients (15.5%) being upstaged to metastatic disease.

**Fig. 1:**
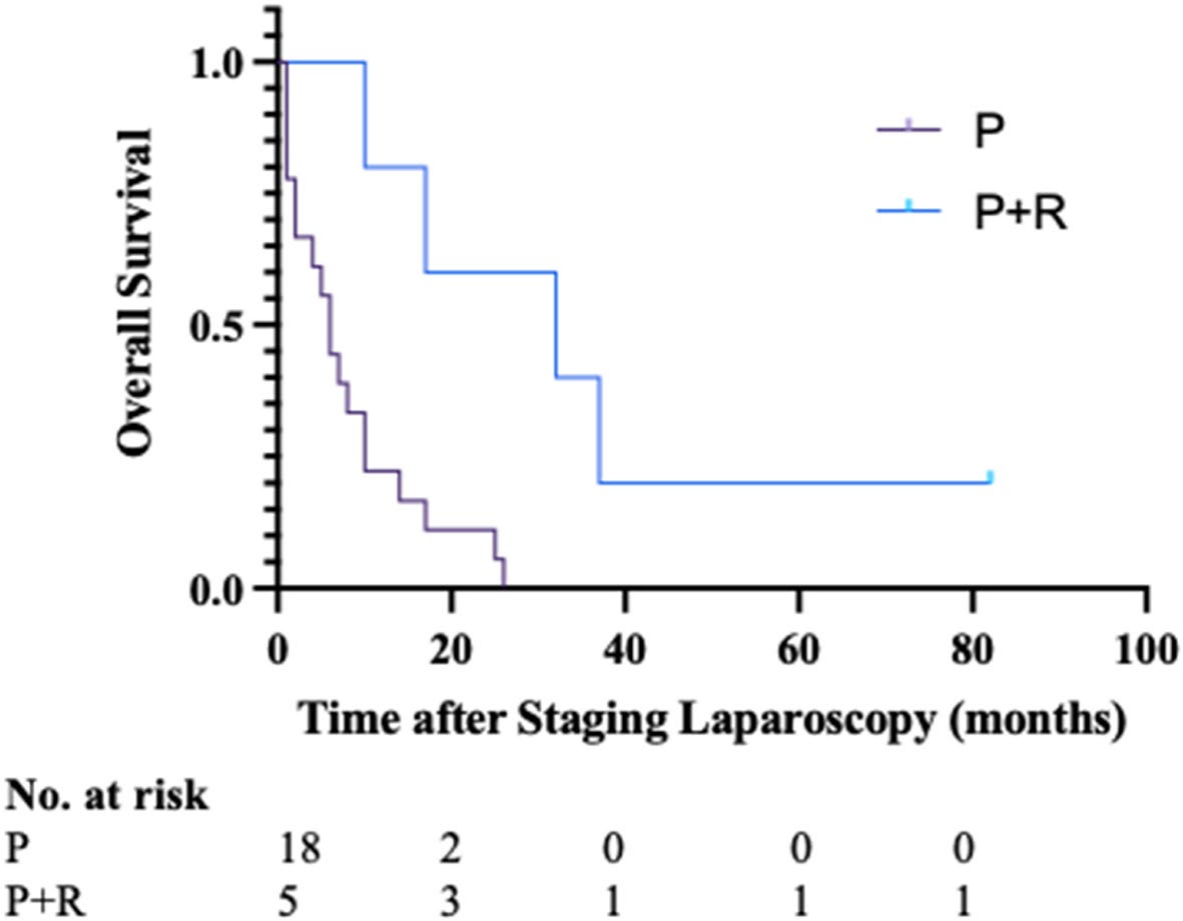
Kaplan–Meier analysis of overall survival of patients after a positive staging laparoscopy that A did not undergo resection and B underwent resection after chemotherapy and a subsequent negative staging laparoscopy. P, positive staging laparoscopy without resection. P + R, positive staging laparoscopy with resection. *P* = 0.003 (log -rank test)

**Fig. 2:**
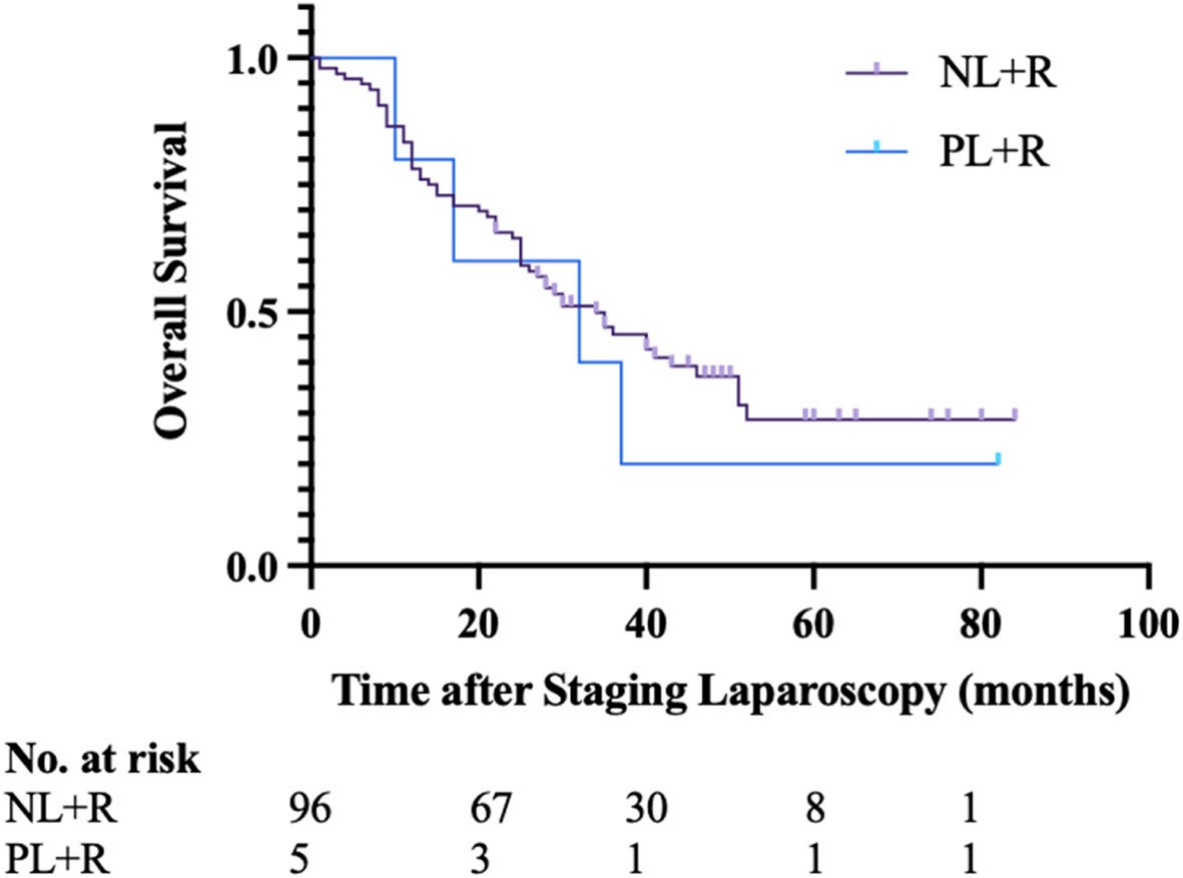
Kaplan–Meier analysis of overall survival of patients undergoing resection- based off staging laparoscopy outcome. NL + R, negative staging laparoscopy with resection. PL + R, positive staging laparoscopy with resection. *P* = 0.642 (log- rank test)

In summary, patients that had no evidence of metastatic disease on preoperative imaging and that subsequently had a positive staging laparoscopy with either macroscopic deposits or positive cytology were upstaged and underwent chemotherapy with ongoing MDT review of the response to chemotherapy. For patients with upfront, borderline and LA disease, the overall survival of the patients that had a positive staging laparoscopy with a subsequent resection was 32 months compared with 6 months for the 19 patients who did not have a resection (Fig. 1, *P* = 0.003) and compared with 34 months for the 96 patients that underwent resection with a negative staging laparoscopy (Fig. 2, *P* = 0.642).

### Management

Across the cohort of 155 patients, 31 underwent upfront surgical resection (20.0%) after staging laparoscopy (median 17 days, range 4- − 74 days after staging laparoscopy), and 67 underwent surgical resection after neoadjuvant chemotherapy (43.2%) (median 135 days, range 40- − 315 days after staging laparoscopy) (Table 4).

**Table 4:** Management of staging laparoscopy patients. Palliative therapy was initiated after a positive staging laparoscopy or due to disease progression while the patient was receiving neoadjuvant chemotherapy

Management	Number
Upfront surgical resection	31 (20%)
Surgical resection after NAC	67 (43%)
Surgery abandoned	3 (2%)
Palliative therapy after positive staging laparoscopy	19 (12%)
Palliative therapy during NAC	35 (23%)

A total of 54 patients received palliative therapy (34.8%). Three patients proceeded to laparotomy but had an abandoned resection due to undiagnosed metastatic disease. All three of these cases were due to the presence of liver peritoneal surface metastases that were undetected on staging laparoscopy.

### Number needed to treat

Staging laparoscopy resulted in 15.5% of patients being upstaged to metastatic disease with a remaining 3.0% having a nontherapeutic laparotomy despite negative staging laparoscopy. Thus, the absolute risk reduction for patients undergoing a staging laparoscopy to avoid a nontherapeutic laparotomy is 12.5%. This produces a number needed to treat value of 8.

## Discussion

Staging laparoscopy continues to have a significant role in the preoperative assessment of PDAC [7–9]. At the Royal North Shore Hospital, 14 patients with either upfront resectable or borderline resectable PDAC had a positive staging laparoscopy (12.2%). Staging laparoscopy was of value for 10 of these patients by avoiding an unnecessary laparotomy by diagnosing macroscopic disease, which would have been noted at the time of attempt for resection. Also, four patients avoided non-curative resection through the finding of microscopic disease at the laparoscopy. This unit’s nontherapeutic laparotomy rate was 3.0%, due to hepatic metastatic deposits not identified on imaging or laparoscopy. While NCCN guidelines suggest MRI as a helpful adjunct to CT staging [3], it is not routinely used at this institution. New intraoperative imaging modalities have emerged to further enhance the utility of staging laparoscopy. Oba et al. (2021) [10] utilised intraoperative ultrasound and fluorescence imaging during staging laparoscopy and detected occult liver metastases in an additional six of the 31 patients.

The NCCN guidelines [3] recommend selective use of staging laparoscopy in pancreatic adenocarcinoma patients who are considered to be high risk for occult metastatic disease including high CA19.9 (> 100 U/mL [11]) and patients with borderline resectable tumours. In their meta-analysis, De Rosa et al. (2016) found CA19-9 ≥ 150 U/Ml and tumour size > 3 cm to be the strongest predictors of unresectable disease [12]. However, this study found no correlation in the outcome of the staging laparoscopy and with the serum CA19-9, tumour size or location.

NCCN guidelines do not comment on the utility of peritoneal washing cytology [3]. Recent studies have shown that patients with positive cytology that undergo resection have poorer outcomes than cytology-negative patients. Ferrone et al. [13] found the median survival of its 10 patients undergoing resection in the setting of positive cytology to be 8 months compared to 16 months for the remaining 208 resected patients with negative cytology. Similarly, Aoki et al. [14] in their study found that the 123 resected patients who were cytology negative after neoadjuvant therapy had significantly better survival than the 13 resected patients that were cytology positive after neoadjuvant therapy (30.8 months vs 14.8 months, respectively). This study is one of the few in the world to report on patients who have had positive cytology, neoadjuvant chemotherapy leading to conversion to negative cytology with favourable outcomes.

There were five patients that had positive macroscopic disease without positive cytology in the peritoneal washings collected. This highlights the imperfect sensitivity of morphologic observation of the peritoneal washings in detecting occult metastases. Recent studies have described the prognostic relevance of microRNA and DNA PCR testing in patients treated for pancreatic ductal adenocarcinoma which could compliment cytology assessment [15]. In their study of 89 patients, Suenaga et al. [16] found that patients with high levels of peritoneal tumour DNA had significantly poorer disease-free survival. Kubo et al. [17] found higher expression of microRNA (miRNA 194-5p) in peritoneal washings to be associated with peritoneal recurrence after radical resection.

We acknowledge the limitations of this study. The follow-up for patients with the most recent staging laparoscopy at the end of 2019 was limited to a follow-up period of 2 years.

We acknowledge that longer follow-up than 2 years is necessary to identify the true value of neoadjuvant therapy for patients with positive cytology. Furthermore, this study is limited to a single high-volume referral centre. We believe this data are convincing enough to warrant a prospective multi-institutional trial with rigid enrolment criteria and methods of care to further explore the utility of neoadjuvant chemotherapy followed by surgical resection in patients that convert to negative staging laparoscopy.

## Conclusion

Staging laparoscopy continues to be a valuable investigation in the staging of potentially operable pancreatic adenocarcinoma. In this institution, one in every eight patients undergoing a staging laparoscopy was upstaged to metastatic disease, thus avoiding an unnecessary laparotomy or a non-curative resection.

## Data Availability

Raw data were generated at the Royal North Shore Public and Royal North Shore Private Hospitals. Derived data supporting the findings of this study are available from the corresponding author (MJ) on request.

## Abbreviations

PDAC: Pancreatic ductal adenocarcinoma
CT: Computed tomography
NCNN: National Comprehensive Cancer Network
PET: Positron emission tomography
MDT: Multidisciplinary team
SUVmax: Maximum standardised uptake values
LA: Locally advanced
GOJ: Gastroesophageal junction
